# Inflammation Promotes Expression of Stemness-Related Properties in HBV-Related Hepatocellular Carcinoma

**DOI:** 10.1371/journal.pone.0149897

**Published:** 2016-02-26

**Authors:** Te-Sheng Chang, Chi-Long Chen, Yu-Chih Wu, Jun-Jen Liu, Yung Che Kuo, Kam-Fai Lee, Sin-Yi Lin, Sey-En Lin, Shui-Yi Tung, Liang-Mou Kuo, Ying-Huang Tsai, Yen-Hua Huang

**Affiliations:** 1 Department of Biochemistry and Molecular Cell Biology, School of Medicine, College of Medicine, Taipei Medical University, Taipei, Taiwan; 2 Division of Gastroenterology and Hepatology, Department of Internal Medicine, Chang Gung Memorial Hospital, Chiayi, Taiwan; 3 Department of Pathology, Wan Fang Hospital, Taipei Medical University, Taipei, Taiwan; 4 Department of Pathology, Taipei Medical University Hospital, Taipei Medical University, Taipei, Taiwan; 5 Department of Pathology, School of Medicine, College of Medicine, Taipei Medical University, Taipei, Taiwan; 6 TMU Center for Cell Therapy and Regeneration Medicine, Taipei Medical University, Taipei, Taiwan; 7 School of Medical Laboratory Sciences and Biotechnology, Taipei Medical University, Taipei, Taiwan; 8 Department of Pathology, Chang Gung Memorial Hospital, Chiayi, Taiwan; 9 Department of General Surgery, Chang Gung Memorial Hospital, Chiayi, Taiwan; 10 Division of Pulmonary and Critical Care Medicine, Department of Internal Medicine, Chang Gung Memorial Hospital, Chiayi, Taiwan; 11 Graduate Institute of Medical Sciences, College of Medicine, Taipei Medical University, Taipei, Taiwan; 12 Center for Reproductive Medicine, Taipei Medical University Hospital, Taipei Medical University, Taipei, Taiwan; 13 Comprehensive Cancer Center of Taipei Medical University, Taipei, Taiwan; 14 Ph.D. Program for Translational Medicine, Taipei Medical University, Taipei, Taiwan; University of Hong Kong, HONG KONG

## Abstract

The expression of cancer stemness is believed to reduce the efficacy of current therapies against hepatocellular carcinoma (HCC). Understanding of the stemness-regulating signaling pathways incurred by a specific etiology can facilitate the development of novel targets for individualized therapy against HCC. Niche environments, such as virus-induced inflammation, may play a crucial role. However, the mechanisms linking inflammation and stemness expression in HCC remain unclear. Here we demonstrated the distinct role of inflammatory mediators in expressions of stemness-related properties involving the pluripotent octamer-binding transcription factor 4 (OCT4) in cell migration and drug resistance of hepatitis B virus-related HCC (HBV-HCC). We observed positive immunorecognition for macrophage chemoattractant protein 1 (MCP-1)/CD68 and OCT4/NANOG in HBV-HCC tissues. The inflammation-conditioned medium (inflamed-CM) generated by lipopolysaccharide-stimulated U937 human leukemia cells significantly increased the mRNA and protein levels of OCT4/NANOG preferentially in HBV-active (HBV^+^HBsAg^+^) HCC cells. The inflamed-CM also increased the side population (SP) cell percentage, green fluorescent protein (GFP)-positive cell population, and luciferase activity of OCT4 promoter-GFP/luciferase in HBV-active HCC cells. Furthermore, the inflamed-CM upregulated the expressions of insulin-like growth factor-I (IGF-I)/IGF-I receptor (IGF-IR) and activated IGF-IR/Akt signaling in HBV-HCC. The IGF-IR phosphorylation inhibitor picropodophyllin (PPP) suppressed inflamed-CM-induced *OCT4* and *NANOG* levels in HBV^+^HBsAg^+^ Hep3B cells. Forced expression of OCT4 significantly increased the secondary sphere formation and cell migration, and reduced susceptibility of HBV-HCC cells to cisplatin, bleomycin, and doxorubicin. Taking together, our results show that niche inflammatory mediators play critical roles in inducing the expression of stemness-related properties involving IGF-IR activation, and the upregulation of OCT4 contributes to cancer migration and drug resistance of HBV-HCC cells. Findings in this paper would provide potential targets for a therapeutic strategy targeting on inflammatory environment for HBV-HCC.

## Introduction

Epidemiological and experimental studies have shown that the inflammatory microenvironment is an indispensable participant in the neoplastic process, including development, proliferation, survival, and migration of many cancers [1]. Hepatocellular carcinoma (HCC) is a prototype of inflammation-associated cancer that generally unfolds on a background of chronic hepatitis, irrespective of the triggering etiology [2]. Despite the emerging new therapeutic options for HCC, the overall survival of patients with this common cancer have not improved, and new therapeutic strategies are urgently required [3]. With the paucity of effective therapy for HCC per se, determining the underlying mechanisms involved in the interaction between tumor and inflammatory microenvironment could theoretically enable the development of synergistic therapeutic strategies targeting on niche inflammation [4]. However, the molecular pathways linking inflammation and HCC remain unclear, and studies elaborating the effect of inflammatory cells and inflammatory mediators on hepatocarcinogenesis are inconclusive [2].

The exponential progress in cancer stem cell (CSC) research in the past two decades has held promise for improved cancer treatment strategies [5]. Linkage between the inflammatory microenvironment and the so-called CSCs has been increasingly elucidated [6, 7]. The fluctuating intensity of inflammation can increase the adaptation of cancer cells, leading to the development of CSCs [8]. Tumor-associated macrophages are involved in modulating tumorigenesis and drug resistance of stem cells in non–small-cell lung cancer and colon cancer [9]. Increased octamer-binding transcription factor (OCT) 3/4-positive cells in *Schistosoma haematobium*-associated inflammation support that stem cells participate in inflammation-related carcinogenesis in bladder cancer [10]. Inflammatory cytokine expression is closely associated with the expansion of liver progenitor cells [11]. Furthermore, in mouse HCC models, CD44^+^ HCC progenitor cells could give rise to cancer only when these cells were introduced into a liver with a chronic inflammation background [12]. These observations highlight the causal relationship between inflammation and cancer stemness properties, particularly for HCC.

Although both hepatitis B virus (HBV) and hepatitis C virus (HCV) can cause liver inflammation, they induce hepatocarcinogenesis through different mechanisms, and the upregulation of insulin-like growth factor-I receptor (IGF-IR) signaling is confined to HBV-related HCC (HBV-HCC) [13, 14]. IGF-IR signaling is essential for maintaining stemness-related properties, including OCT4/NANOG expression in human embryonic stem cells [15, 16] and mouse germline stem cells [17]. Our recent study demonstrated that IGF-IR signaling could enhance OCT4 expression in HBV-HCC [14]. The present study investigated the effect of inflammatory mediators on IGF-IR signaling and cancer stemness-related property expression in HCC. Our data revealed that inflammatory mediators enhanced IGF-IR signaling and resulted in the expression of pluripotent transcription factors OCT4 and NANOG. OCT4 overexpression increased the capacity of stemness-related properties (reinforced secondary sphere formation), tumor invasiveness (enhanced cell migration), and drug resistance (reduced susceptibility to cisplatin, bleomycin, and doxorubicin) in HBV-HCC cells. Our results demonstrate the effect of inflammatory mediators on cancer stemness expression and provide potential targets for a therapeutic strategy targeting on inflammatory environment for HBV-HCC.

## Materials and Methods

### Cell lines and HCC tissues

Hep3B (HBV^+^HBsAg^+^ human HCC, HB-8064^™^) and HepG2 (HBV^−^ human hepatoblastoma, HB-8065^™^) cells were purchased from American Type Culture Collection (ATCC, Manassas, VA, USA). Huh7 (HBV^−^ human HCC) cells were obtained from the Japanese Collection of Research Bioresources (Tokyo, Japan). HepG2.2.15 (HBV^+^HBsAg^+^ human hepatoblastoma) cells (Cl33, HyperCLDB, Genova, Italy) and U937 cells (CRL-1593.2, ATCC) were provided by Dr. Jun-Jen Liu, Institute of Medical Biotechnology, College of Medicine, Taipei Medical University, Taipei, Taiwan. HA22T (HBV^+^HBsAg^−^ human HCC) cells (60168, Bioresource Collection and Research Center, Hsinchu, Taiwan) were provided by Dr. Ching-Tai Lin, Chang Gung Memorial Hospital, Chiayi, Taiwan. Cells were maintained in Dulbecco’s Modified Eagle Medium (DMEM) (Gibco-BRL, Grand Island, NY, USA) supplemented with 10% fetal bovine serum (FBS) and 1.5 g/L sodium bicarbonate. The IGF-IR phosphorylation inhibitor picropodophyllin (PPP, Calbiochem, Nottingham, UK) was resuspended in dimethyl sulfoxide to obtain a final concentration of 1 uM. Human HCC tissue sections were obtained from the Tissue Bank, Department of Medical Research, Chang Gung Memorial Hospital at Chiayi, Taiwan. Written informed consent was obtained from all the patients providing HCC tissues. This study was approved by the Institutional Review Board of Chang Gung Medical Foundation (Approval number: 98-2675B).

### RNA isolation and real-time reverse-transcription polymerase chain reaction

HCC cell lines were subjected to total RNA isolation and quantitative real-time reverse-transcription polymerase chain reaction (RT-PCR). For cell lines, total RNA was extracted using an RNeasy Micro Kit (Qiagen, Valencia, CA, USA) according to the manufacturer’s instructions. Frozen tissues were homogenized in liquid N_2_ and lysed in RNA extraction buffer. Three micrograms of total RNA was used to synthesize complementary (c)DNA by using a random primer (Invitrogen, Carlsbad, CA, USA). cDNA synthesis was performed at 42°C for 50 min in a final volume of 20 uL according to the manufacturer’s instructions for Superscript β reverse transcriptase (Invitrogen). PCR was conducted using PlatinumTaq^™^ Polymerase (Invitrogen), and RT-PCR amplifications were titrated to be within a linear range of amplification. For real-time RT-PCR, PCR amplification was performed using the QuantiTect SYBR Green PCR Mix (Qiagen). Primer sequences, annealing temperatures, and PCR cycling conditions are listed in S1 Table. Beta-2M was used as an internal control. Real-time RT-PCR of at least three independent cultures was performed in all experiments.

### Western blotting

Proteins from the human HCC tissues and human HCC cell lines were extracted using lysis buffer containing 1% Triton X-100, 150 mM NaCl, 1 mM EDTA, and 10 mM Tris-HCl (pH 7.5) with a protease inhibitor cocktail (Roche Diagnostics, NA USA). The concentration of these proteins was measured using a BCA protein quantification kit (Pierce, Rockford, IL, USA). Twenty to fifty micrograms of total proteins was boiled in Laemmli buffer, loaded onto a 10–20% sodium dodecylsulfate polyacrylamide gel electrophoresis (SDS-PAGE) minigel, transferred to a polyvinylidene difluoride (PVDF) membrane, and subjected to Western blotting. We used a mouse monoclonal anti-OCT4 antibody (sc-5279, Santa Cruz Biotechnology), anti-NANOG antibody (ab21624, Abcam), anti-IGF-IRβ antibody (sc-713, Santa Cruz Biotechnology), anti-EGFP antibody (sc-9996, Santa Cruz Biotechnology), and anti-β-actin antibody (sc-47778, Santa Cruz Biotechnology) as the primary antibodies and a HRP-conjugated goat anti-rabbit/or mouse IgG (Jackson ImmunoResearch) as the secondary antibody (S2 Table). In brief, PVDF membranes were blocked with 5% skim milk in Tris-buffered saline (TBS) for 1 h at room temperature and incubated with the primary antibodies at 4°C overnight. After washing with TBS containing 0.05% Tween-20 (v/v; TBST), blots were incubated with the secondary antibody at a dilution of 1:5000 at room temperature for 1 h. After washing four times with TBST, the immunoreactive bands were developed using an enhanced chemiluminescence system (Amersham Pharmacia Biotech, Buckinghamshire, UK).

### Immunohistochemical staining

Paraffin-embedded HCC tissues (5-μm-thick sections) were deparaffinized using xylene and rehydrated in a graded alcohol series. Thereafter, for immunorecognition, tissue sections mounted on slides were heated in 0.01 M citric buffer (pH 6.0) for 10 min with autoclaving. After cooling down to room temperature, sections were treated with 3% H_2_O_2_ in phosphate-buffered saline (PBS) at room temperature for 30 min. Slides were washed and blocked with 5% normal horse serum in PBS and incubated with the primary antibodies antimacrophage chemoattractant protein 1 (anti-MCP-1) (sc-1784, Santa Cruz Biotechnology, Santa Cruz, CA, USA), anti-CD68 (ab955, Abcam, Cambridge, UK), and control IgG (no. 76870, Jackson ImmunoResearch, West Grove, PA, USA) (S2 Table) at 4°C overnight, followed by incubation with its related horseradish peroxidase (HRP)-conjugated secondary antibody. HRP enzyme activity was detected using a labeled streptavidin-biotin (LSAB) system with 3,3’-diaminobenzidine tetrachloride as the chromogen according to the manufacturer’s instructions (DakoCytomation, Carpentaria, CA, USA). Nonspecific binding sites were blocked using Protein Block (DakoCytomation). The immunostained sections were counterstained using hematoxylin and were dehydrated and mounted. Sections were evaluated by Image Scope software (Aperio Technologies, Vista, CA, USA) for quantitative analysis. The number of CD68 positive cell was counted. The intensities of positive staining of OCT4, NANOG, and MCP1 were presented as the total intensity per total pixels. Three randomly selected high-power fields were analyzed for each tumor section.

### Generation of inflammation-conditioned medium

The inflammation-conditioned medium (inflamed-CM) was generated using lipopolysaccharide (LPS, Sigma-Aldrich, St. Louis, MO)-stimulated U937 human leukemia cells. In brief, U937 cells were reseeded at a density of 6.3 × 10^4^ cells/cm^2^ in 10% FBS-DMEM and were stimulated with LPS (1 ug/mL) for 4 days. The inflamed-CM was obtained from the growth medium after centrifugation column separation for removal of cell debris and any remaining LPS, respectively. The purified human inflamed-CM was then stored at −80°C until use.

### Plasmids and dual luciferase assay

The promoter OCT4-green fluorescent protein (GFP) plasmids, retrovirus-packaged pMXs-enhanced green fluorescence protein (EGFP), and pMXs-OCT4 plasmids were provided by Dr. Hung-Chih Kuo at the Stem Cell Program, Academia Sinica, Taipei, Taiwan. The promoter OCT4-GFP plasmids and control vectors (pRL-TK, Promega, Madison, WI, USA) were co-transfected into HepG2 and HepG2.2.15 cell lines at a molar ratio of 10:1 by using Turbofect reagent according the manufacturer’s instructions (R0531, Fermentas, York, UK). The OCT4 promoter luciferase-HepG2215 cell line was generated by transfection of HepG2.2.15 with OCT4 promoter-luciferase plasmids (from Dr. Muh-Hwa Yang, National Yang-Ming University, Taipei, Taiwan) and control vectors pRL-TK at a molar ratio of 1:10. Luciferase activity was measured according to the manufacturer’s protocol. Data are represented as the ratio of firefly to *Renilla* luciferase activity.

### Cell viability assay

For the proliferation assay, pMXs-EGFP or pMXs-OCT4 virus-infected Hep3B cells were seeded in 96-well plates at 10^4^ cells/well and incubated at 37°C in 5% CO_2_ for 24, 48, or 72 h. For the drug sensitivity assay, the cells were seeded for 24 h and treated with various concentrations of cisplatin (P4394, Sigma-Aldrich), bleomycin (Bleo TM, Nippon Kayaku, Tokyo), or doxorubicin (DOX, adriamycin, Actavis Italy SpA, Beijing, China), and these cells were then incubated at 37°C in 5% CO_2_ for 48 h. Thereafter, a WST-1 assay (Roche) was used to detect cell proliferation according to the manufacturer’s instructions. Three experiments were performed for each experimental condition. Cell viability is expressed as the percentage of non-treated cells.

### Transwell migration assays

Transwell assays were performed using 8-μm pore transwell chambers in 24-well plates (Corning Costar, Cambridge, MA, USA). The upper chambers were seeded with 1 × 10^5^ Hep3B cells in 100 uL of the serum-free DMEM/F12 medium. These Hep3B cells had been previously transfected with either the control pMXs-EGFP vector or the pMXs-OCT4 plasmid. The lower chambers were filled with 800 uL of the DMEM/F12 medium containing 10% FBS. Subsequently, the cells were incubated at 37°C in a 5% CO_2_ humidified atmosphere for 24 h. After swabbing the upper chambers to remove cells that did not migrate, the cells that migrated to the lower chambers were fixed with 3.7% paraformaldehyde in PBS and stained using hematoxylin. The migrated cells were counted under a light microscope in five predetermined fields. The assays were performed in triplicate, and the results are expressed as the percentage of the mean of three wells containing cells transformed with the control vectors.

### Secondary tumor sphere formation assay

The pMXs-EGFP or pMXs-OCT4 virus-infected HepG2 cells were used for the sphere formation assay. Tissue culture dishes were coated with polyhydroxyethyl methacrylate polymer (polyHEMA, Sigma-Aldrich) to facilitate sphere formation. A 12% polyHEMA solution was prepared in 95% ethanol, and 0.5 mL of a 1:10 dilution of this solution (in 95% ethanol) was added to each well of a six-well plate. A hydrophobic surface was created as the polyHEMA solution dried at room temperature in a tissue culture hood. The pMXs-EGFP or pMXs-OCT4 virus-infected HepG2 cells were plated at a density of 1000 cells/well in the DMEM-F12 medium (Invitrogen, Carlsbad, CA, USA) containing 2% B27 supplement (Invitrogen). Media were replenished every 2–3 days. Methyl cellulose (1%, Sigma-Aldrich) was added to prevent cell aggregation, and the individual spheres derived from single cells were confirmed. The spheres were harvested after culturing for 10 days and were dissociated into single cells by treatment with trypsin for the secondary sphere formation. After 7 days, spheres with diameter greater than 75 μm were photographed and counted.

### Flow cytometry of side population cells

To examine the percentage of side population (SP) cells, HCC cells with or without 24 h inflamed-CM treatment were sorted through FACS flow cytometry (FACS Vantage, Becton Dickinson, San Jose, CA, USA). In brief, HCC cells were trypsinized and incubated at 4°C for 30 min with Hoechst red and Hoechst blue (Cell Signaling Technology, Danvers, MA). The nonspecific fluorescence signals were excluded using an antiporter-specific inhibitor, FIC. Labeled cells were analyzed and separated using a cell sorter (FACS Vantage, Becton Dickinson). The percentage of SP cells was determined by subtracting the percentage of parent (H + FTC) from the percentage of parent (H) {(% of SP = % of parent (H) − % of parent (H + FTC))}. Collected data were analyzed using CELLQuest software (Becton-Dickinson Immunocytometry Systems, San Jose, CA, USA). Gating was implemented based on negative-control staining profiles. SP cells were seeded in culture dishes for additional experiments.

### Statistical analyses

All experiments were repeated at least three times. The results are presented as the mean ± the standard deviation (SD) as appropriate, and were analyzed using the Student *t* test. The Spearman’s correlation analysis was used to examine the correlation between the CD68/MCP1 and OCT4/NANOG. *P* < .05 was considered statistically significant.

## Results

### Expression of MCP-1/CD68 and pluripotent transcription factors OCT4 and NANOG in HBV-HCC tissue

**Fig 1:**
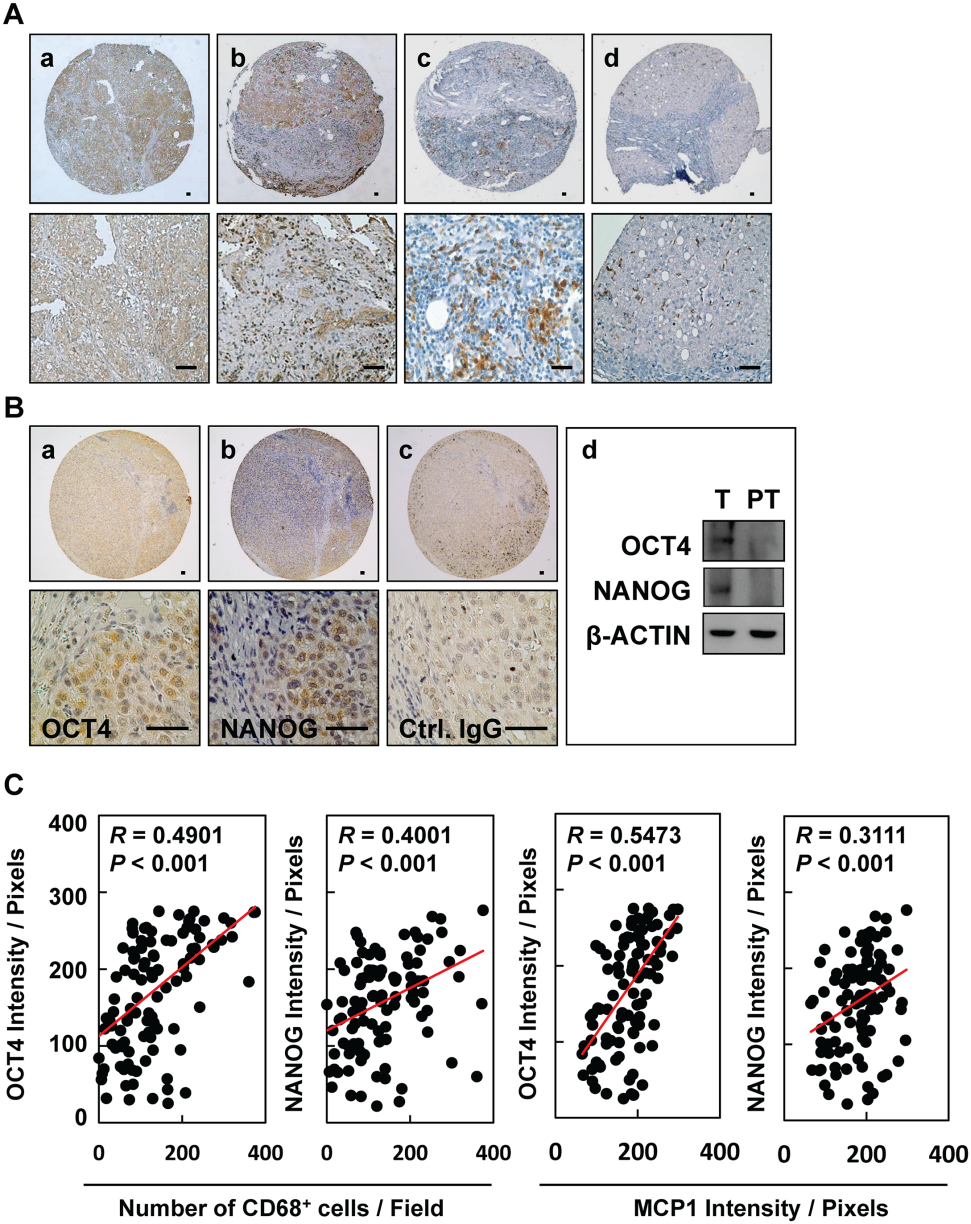
Expression of MCP-1/CD68 and pluripotent transcription factors OCT4 and NANOG in HBV-HCC tissues. Immunohistochemical staining of **(A)** MCP-1 (a, b) and CD68 (c, d) and **(B)** OCT4 (a) and NANOG (b) in HBV-HCC tissues. (d) Expression of OCT and NANOG in tumor (T) and peritumor (PT) tissues from patients. Bar = 50 um. **(C)** The correlations between the inflammatory levels (CD68 and MCP1) and the protein levels (OCT4 and NANOG) in HCC patient tissues were shown (*n* = 129).

To examine whether inflammation provides a niche environment for the expression of stemness-related genes in HBV-HCC tissues, immunohistochemical staining of inflammation-associated proteins was performed. We observed positive immunorecognition of antibodies against MCP-1 (Fig 1A, panels a and b) and CD68 (Fig 1A, panels c and d) in HBV-HCC tissues. HBV-HCC tissues showed positive immunosignals for pluripotent transcription factors OCT4 and NANOG, as demonstrated by immunohistochemical staining (Fig 1B, panels a and b) and Western blotting (Fig 1B, panel d). Control IgG served as a negative control (Fig 1B, panel c). There are significantly positive correlations between the inflammatory levels of CD68/MCP1 and the protein levels of OCT4/NANOG in HCC tissues (Fig 1C, *P* < 0.001, *n* = 129). There is a higher significantly positive association between CD68/MCP1 and OCT4 in HBV-HCC (S1A Fig) when compared to that of HBV-negative HCC (S1B Fig). These results highlight that niche immune cell infiltration and inflammatory stimulation may be associated with the expression of OCT4/NANOG in HCC.

### Inflamed-CM increased OCT4/NANOG expression and SP cell percentage in HBV-HCC cell lines

**Fig 2:**
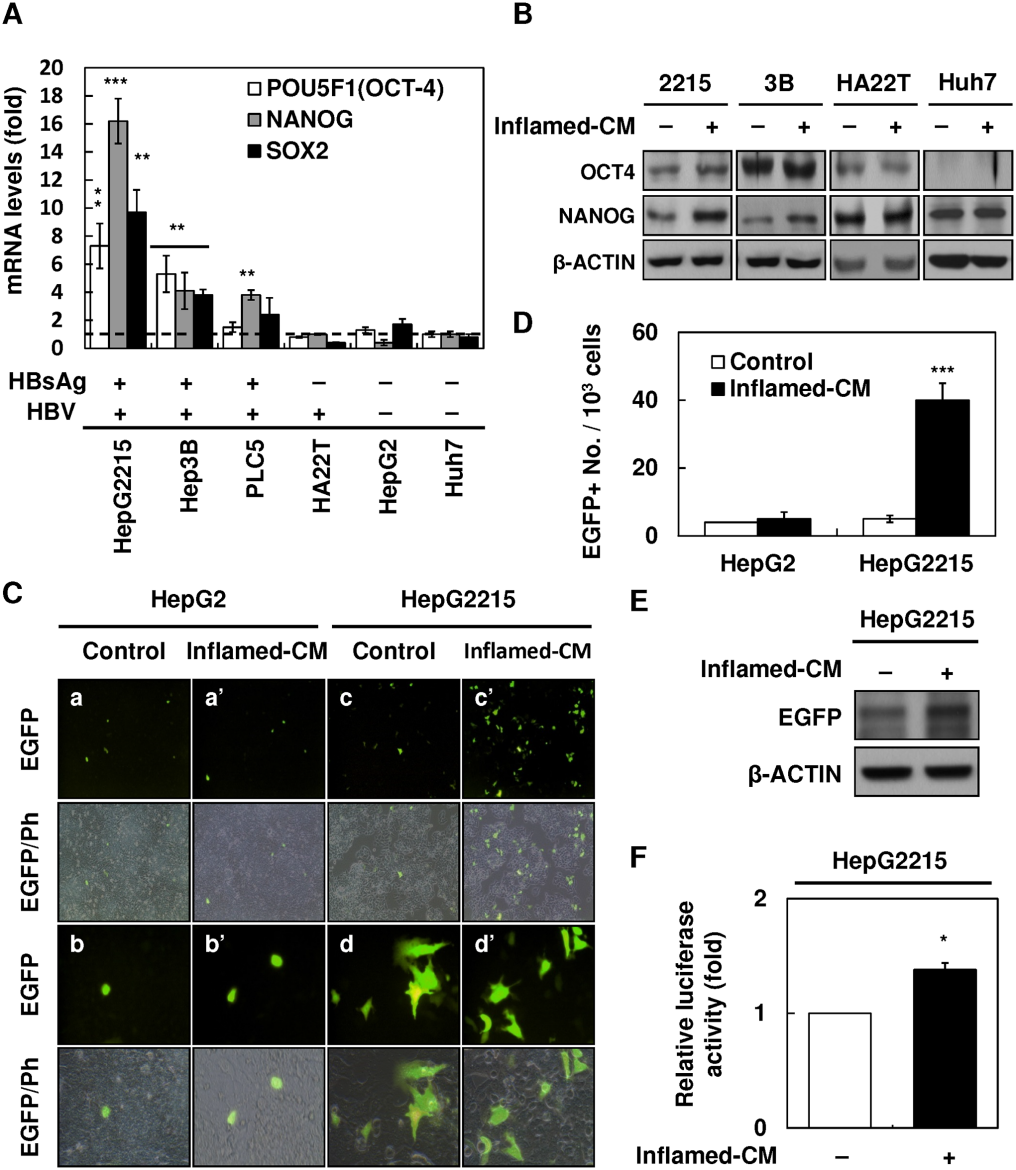
Upregulation of OCT4/NANOG expression in HBV-related hepatocellular carcinoma (HBV-HCC) cell lines with inflammation-conditioned medium (inflamed-CM) treatment. **(A)** mRNA levels of stemness-related genes (*NANOG*, *OCT4*, and *SOX2*) in human HCC cell lines of HepG2.2.15, Hep3B, PLC5 (HBV^+^HBsAg^+^), HA22T (HBV^+^HBsAg^−^), HepG2, and Huh7 (HBV^−^HBsAg^−^) with inflamed-CM treatment for 7 days (by quantitative real-time RT-PCR). The dashed line indicates gene expression in HCC cells without inflamed-CM treatment (multiple of expression = 1, control group). **(B)** OCT4 and NANOG levels in HCC cells with or without inflamed-CM treatment (through Western blotting). **(C)** Number of EGFP^+^ cells among OCT4 promoter-EGFP HepG2 and HepG2.2.15 cells with and without inflamed-CM treatment. EGFP^+^ HepG2.2.15 cells show mesenchymal-like cell morphology. EGFP, enhanced green fluorescence protein; Ph, phase image. **(D)** Number of EGFP^+^ cells (per 10^3^ cells) among HepG2 and HepG2.2.15 cells with and without inflamed-CM treatment. **(E)** The EGFP level in HepG2.2.15 cells with inflamed-CM treatment (by Western blotting). **(F)** The relative luciferase activity of OCT4 promoter-luciferase HepG2.2.15 cells with inflamed-CM treatment. *P < .05, **P < .01, ***P < .001, by *t*-test.

To examine the effects of inflammatory stimulation on the expression of stemness-related proteins in HCC, HBV-active (HBV^+^HBsAg^+^, HepG2.2.15, Hep3B, and PLC5), HBV-inactive (HBV^+^HBsAg^−^, HA22T), and non-HBV HCC (HBV^−^HBsAg^−^, HepG2, and Huh7) cell lines were treated with optimal concentrations of LPS-stimulated U937-conditioned medium (i.e., inflamed-CM) for 7 days (S2 Fig). As shown in Fig 2, a significant increase in *OCT4*/*NANOG*/*SOX2* mRNA levels was detected in HBV^+^HBsAg^+^ cells (Hep2.2.15 and Hep3B), but not in HBV^+^HBsAg^−^ (HA22T) or HBV^−^HBsAg^−^ (HepG2 and Huh7) cells (the dashed line denotes cells without inflamed-CM treatment, which served as the basal control) (Fig 2A). Furthermore, western blotting confirmed the upregulation of OCT4 and NANOG expression in inflamed-CM-treated HBV^+^HBsAg^+^ HepG2.2.15 and Hep3B cells (Fig 2B). In addition, the fact that inflamed-CM increased OCT4 expression was further supported in the OCT4 promoter-EGFP reporter system. As shown in Fig 2C, the inflamed-CM increased the EGFP levels in HBV^+^HBsAg^+^ HepG2.2.15 cells, but not in HBV^-^HBsAg^-^ HepG2 cells. Notably, EGFP^+^ HepG2.2.15 cells showed mesenchymal-like morphology when compared with that of GFP^−^ HepG2.2.15 cells (Fig 2C, panels d and d'). The effect of the inflamed-CM on upregulating OCT4 expression in Hep2.2.15 was further demonstrated by the significantly higher number of EGFP^+^ cells (Fig 2D), higher EGFP levels (Fig 2E), and increased OCT4-promoter-luciferase activity (Fig 2F). In addition, the inflamed-CM increased the percentage of SP cells in HBV^+^HBsAg^+^ HepG2.2.15 and Hep3B cells, but not in HBV-inactive HA22T or HBV^−^HBsAg^−^ HepG2 and Huh7 cells (Table 1). These results support that inflammatory mediators promote the expression of stemness-related proteins such as OCT4/NANOG preferentially in HBV-HCC cells.

**Table 1:** Inflammatory effect on SP population in human HCC cell lines.

HCC	Characteristics	HBV/HBsAg	Cell type	SP (%)	SP-inflamed-CM (%)
HepG2215	Hepatoblastoma	+/+	Epithelial	2.4	3.0
Hep3B	HCC	+/+	Epithelial	0.4	1.4
PLC-8024	HCC	+/+	Epithelial	0.2	0.4
HA22T	HCC	+/−	Epithelial	0.0	0.0
HepG2	Hepatoblastoma	−/−	Epithelial	0.1	0.1
Huh7	HCC	−/−	Epithelial	0.1	0.2

HCC: hepatocellular carcinoma; SP: side population

### Upregulation of IGF-I/IGF-IR and IGF-IR/Akt signaling in HBV-HCC cell lines with inflamed-CM treatment

**Fig 3:**
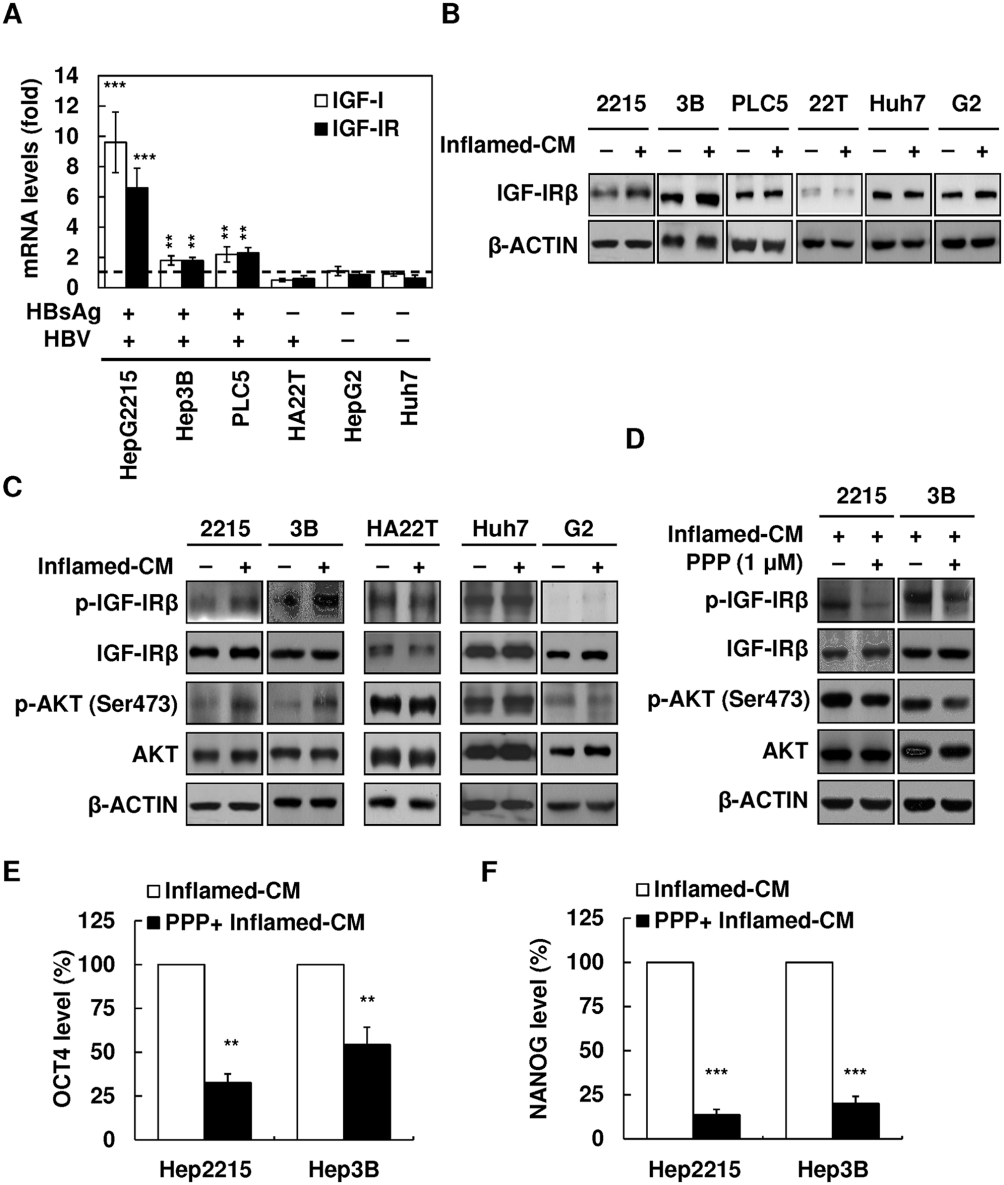
Upregulation of IGF-I and IGF-IR in HBV-HCC cell lines with inflamed-CM treatment. mRNA levels of *IGF-I* and *IGF-IR* in human HCC cell lines of HepG2.2.15, Hep3B, PLC5 (HBV^+^HBsAg^+^), HA22T (HBV^+^HBsAg^−^), HepG2, and Huh7 (HBV^−^HBsAg^−^) with inflamed-CM treatment for 7 days (by quantitative real-time RT-PCR). The dashed line indicates the gene expression in HCC cells without inflamed-CM treatment (multiple of expression = 1, control group). **(B)** IGF-IR levels in HCC cells with and without inflamed-CM treatment (by Western blotting). **(C)** Effect of inflamed-CM on the activation of IGF-IR/Akt signaling in HBV^+^HBsAg^+^ HepG2.2.15 and Hep3B is shown. **(D)** Effect of IGF-IR phosphorylation inhibitor PPP on inflamed-CM-induced IGF-IR/Akt signaling activation is shown. Effect of PPP (1 μM) on inflamed-CM-induced mRNA levels of *OCT4* **(E)** and *NANOG* **(F)** in HepG2.2.15 and Hep3B cells is shown. **P < .01, ***P < .001, by *t*-test.

The upregulation of IGF-IR/Akt signaling is shown to express preferentially in HCCs with an HBV etiology [13]. We determined whether inflammatory stimulation enhanced the expression of IGF-I and IGF-IR in HBV-HCC. As shown in Fig 3, treatment with the inflamed-CM for 7 days significantly increased both mRNA and protein levels of IGF-I and IGF-IR in HBV^+^HBsAg^+^ HepG2.2.15 and Hep3B cells (Fig 3A and 3B, real-time quantitative RT-PCR and western blotting). Meanwhile, the inflamed-CM markedly increased the levels of phospho-IGF-IR (p-IGF-IR) and phospho-AKT (p-AKT), particularly in HepG2.2.15 and Hep3B cells (Fig 3C). The IGF-IR phosphorylation inhibitor PPP effectively suppressed the inflamed-CM-induced p-IGF-IR and p-Akt expression in HepG2.2.15 and Hep3B cells (Fig 3D). PPP also effectively suppressed the inflamed-CM-induced increase in the mRNA levels of *OCT4* (Fig 3E, *P* < .01) and *NANOG* (Fig 3F, *P* < .001). These results support the role of inflammation-mediated IGF-IR signaling in OCT4/NANOG expression in HBV-HCC.

### Forced expression of OCT4 promoted cell migration and reduced drug susceptibility of HBV-HCC cells

**Fig 4:**
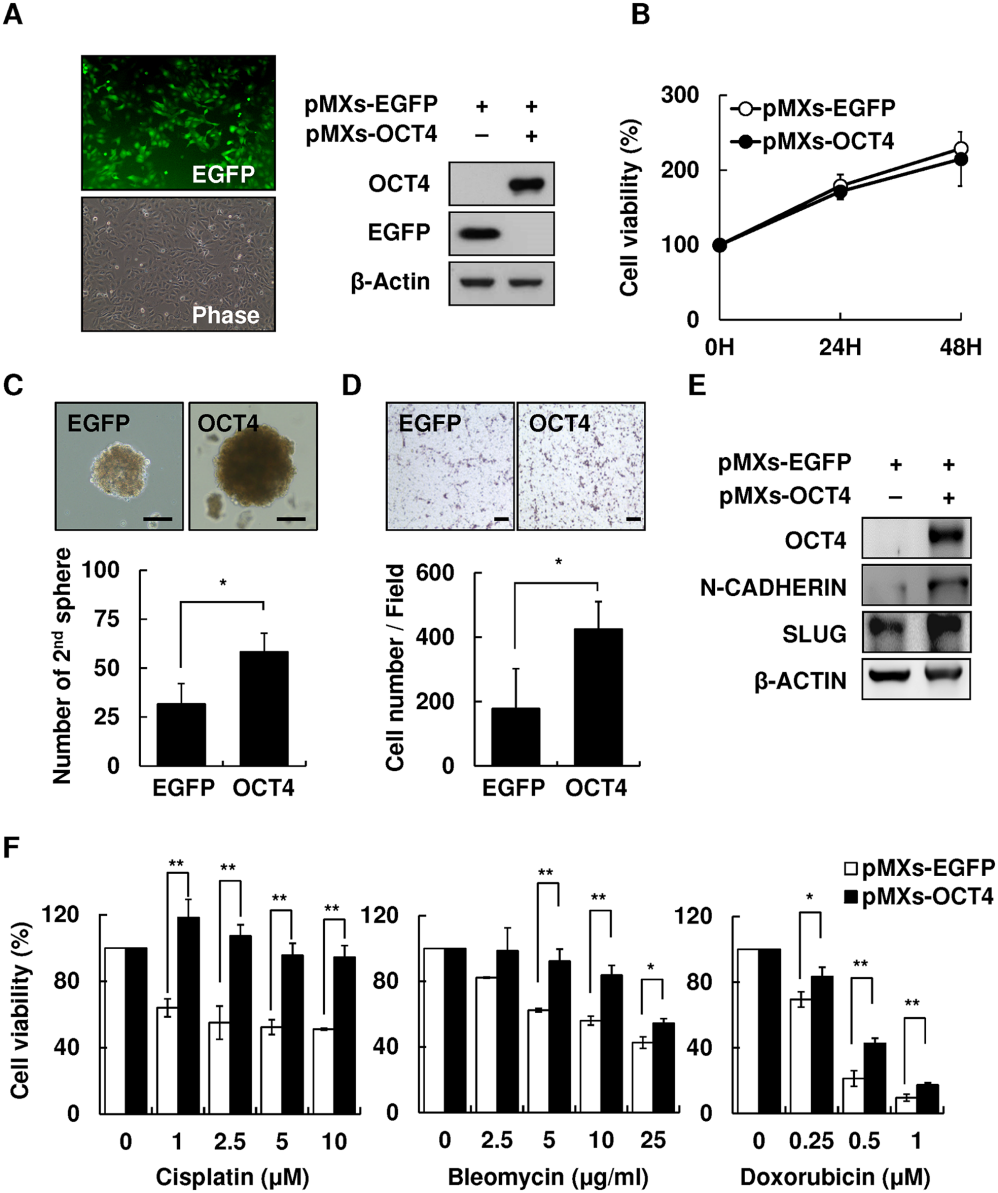
Overexpression of OCT4 increased secondary sphere formation and cell migration and reduced drug susceptibility of HCC cells. Overexpression of pMXs-OCT4 in Hep3B cells. **(B)** Cell proliferation assay of control pMXs-EGFP and pMXs-OCT4 Hep3B for 24, 48, and 72 h. **(C)** The secondary sphere formation percentage of control pMXs-EGFP and pMXs-OCT4 Hep3B under non-adhesion assay. Bar = 100 um. **(D)** Transwell assay of control pMXs-EGFP and pMXs-OCT4 Hep3B. Bar = 100 um. **(E)** The expression levels of migration-related protein N-cadherin and Slug in control pMXs-EGFP and pMXs-OCT4 Hep3B. **(F)** The cell viability of control pMXs-EGFP and pMXs-OCT4 Hep3B cells after treatment with cisplatin (0, 1, 2.5, 5, and 10 μM), bleomycin (0, 2.5, 5, 10, and 25 ug/mL), or doxorubicin (0, 0.25, 0.5, and 1 uM). *P < .05, **P < .01, by *t*-test.

To examine the effect of OCT4 on cell migration and drug susceptibility of HBV-HCC cells, Hep3B cells were transfected with pMXs-OCT4 or control pMXs-EGFP plasmids to generate cells overexpressing OCT4. As shown in Fig 4A, the transfection efficiency was approximately 70–80% according to the percentage of EGFP^+^ Hep3B cells. Western blotting confirmed OCT4 overexpression in transfected Hep3B cells. No difference was observed in the cell proliferation rate between pMXs-OCT4 and pMXs-EGFP Hep3B cells within 72 h incubation (Fig 4B). The OCT4-overexpressing pMXs-EGFP Hep3B cells showed augmented stemness-related properties, including the secondary sphere formation (Fig 4C) and cell migration, as demonstrated by the transwell assay (Fig 4D) and western blotting (Fig 4E). Furthermore, because the re-expression of stemness-related properties in cancer cells has been clearly implicated to reduce susceptibility to chemotherapeutic drugs [18], we examined the effect of forced expression of OCT4 on drug sensitivity. pMXs-OCT4- and pMXs-EGFP Hep3B cells were treated with three most commonly used chemotherapeutic drugs for HCC, cisplatin, bleomycin, and doxorubicin, for 48 h. As shown in Fig 4F, pMXs-OCT4 Hep3B cells showed a significantly lower susceptibility to cisplatin, bleomycin, or doxorubicin in comparison with that shown by control pMXs-EGFP Hep3B cells (*P* < .01).

These results demonstrated that niche inflammatory stimulation promoted IGF-IR/Akt signaling and OCT4/NANOG expression. The inflammation-induced upregulation of OCT4 increased cell migration and reduced drug susceptibility of HBV-HCC cells.

## Discussion

Researches have established that the inflammatory microenvironment is critical in modulating the pathogenesis of liver diseases, including hepatocarcinogenesis [2]. Because the tumor microenvironment comprises complex components including at least various cells, growth factors, proteolytic enzymes, and inflammatory cytokines, the cross talk between HCC cells and their surrounding microenvironment is difficult to clarify. In addition, each component of the tumor microenvironment may share some functional redundancies; hence, targeting a specific component of the tumor microenvironment may not necessarily interfere with HCC progression [19]. Using the inflamed-CM generated from LPS-stimulated U937 human leukemia cells, our study focused on the interaction between noncellular components, particularly inflammatory cytokines and HCC. We demonstrated that the inflamed-CM altered HCC behavior by modulating cancer stemness-related properties through IGF-IR/Akt signaling in HCC. Intriguingly, the upregulation of IGF-IR and stemness-related properties preferentially occurred in HBV-HCC, suggesting a distinct synergistic effect between HBV and inflammatory cytokines.

The inflamed-CM used in this study was generated from stimulation of immune cells by LPS, a product of gram-negative bacteria. Consequently, the inflammatory mediators in the inflamed-CM may not be completely responsible for liver inflammation, which is usually elicited by the hepatitis virus or hepatotoxic chemicals such as ethanol. Moreover, because the human hepatitis virus cannot infect mice or rats, a rodent model of virus induced hepatitis is currently unavailable. Furthermore, the cell culture models of virus infection, including infection by hepatotropic viruses, cannot be satisfactorily manipulated [20]. Because both *in vitro* and *in vivo* models of liver cancer pathogenesis are limited by their restricted host range and hepatotropism, LPS-induced inflammation is an acceptable alternative for investigating the mechanisms of inflammation-induced hepatopathogenesis [21].

Gene expression profiling of HCC has shown that HBV and HCV induce hepatocarcinogenesis through different mechanisms [13]. Our results suggest that inflammation precipitates in HCC development by evoking diverse etiologies through different mechanisms. Because HCV-HCC patients usually have a higher hepatitis activity index, the risk of tumor recurrence after hepatectomy in HCV-HCC patients is widely believed to be higher than that in HBV-HCC patients [22, 23]. Our study suggests that the difference in the expression levels of “cancer stemness” between HBV-HCC and HCV-HCC might partially explain the clinical observation that HBV-HCC patients have an earlier postoperative tumor recurrence in some circumstances [14, 24]. We found that the inflammatory niche increased IGF-1R and OCT4/NANOG expression preferentially in HBV-HCC cells, particularly in cells actively secreting HBsAg. This observation highlights the critical role of HBsAg in cancer stemness-related properties expression and provides partial explanation for the enhanced oncogenic effects of retained HBsAg in the endoplasmic reticulum when co-expressed with HBV X protein as shown in a recent study [25].

Over the past few years, accumulating evidence has supported that many solid tumors including HCC exhibit stemness properties [26]. Stemness markers NANOG, OCT4, SOX2, c-MYC, and KLF-4 are transcription factors essential for the maintenance of the pluripotency of stem cells [27], and some of them are frequently upregulated in tumors [14, 18, 28]. Tumor cells expressing one or more of these pluripotent transcription factors are believed to represent CSCs. Substantial progress has also been made in identifying cell populations with CSC characteristics in HCC. These characteristics include the expression of various stemness markers and the existence of SP cells [29]. The latter characteristic also represents the existence of CSCs that possess a unique drug resistance capacity, which is attributed to the ability of SP cells to efflux cytotoxic substances as the result of a significant overexpression of ABCG2 transporters [18]. Because CSCs are responsible for immortality, resistance to therapy, transplantability, and recurrence of tumors, upregulation of stemness markers may in part explain the worse prognosis of HBV-HCC patients.

However, the precise mechanisms of CSC regulation remain unclear. The close correlation between IGF-1R signaling and stem cell markers in HBV-HCC in our study suggests that HBV and proinflammatory cytokines are collaboratively involved in CSC formation. In the OCT4/GFP reporter assay using HBV^+^HBsAg^+^ HepG2.2.15 cells, we found the inflamed-CM significantly increased OCT4/NANOG levels (Fig 2A and 2B), and blocking IGR-1R phosphorylation abolished the proinflammatory cytokine-induced enhancement of *OCT4*/*NANOG* mRNA levels in Hep3B cells (Fig 3). Notably, the inflamed-CM also induced the cells to exhibit mesenchymal-like morphology (Fig 2C). This phenomenon supports that the forced expression of OCT4 increases cell migration (Fig 4D) and implies the critical role of the inflammatory niche in CSC maintenance in HBV-HCC.

Characteristic alterations such as the increased expression of IGF-1R have emerged as a crucial event in malignant transformation and the growth of HCC and as a therapeutic target [30]. The cross talk between the different signaling pathways and additional tumor-relevant factors may explain the suboptimal effect of current systemic treatment strategies for HCCs [19]. This may also explain the relative effectiveness of sorafenib, a multikinase inhibitor of the vascular endothelial growth factor receptor, the platelet-derived growth factor receptor, and Raf against HCC [31].http://www.nejm.org/toc/nejm/359/4/ HCC with different etiologies has different molecular characteristics and mechanisms of tumorigenesis [13, 32]. However, the current treatment strategy for HCC does not consider the distinctive underlying mechanism evoked by various etiologies. This is partially because of the paucity of the understanding of the molecular diversity of HCC tumorigenesis. Determining the specific molecular pathways linking the inflammatory reaction and HCC will enable the development of improved therapies for HBV-HCC.

HCC has a heterogenous molecular pathogenesis that involves the activation of complex signaling pathways [33]. Our finding that inflammation-mediated IGF-IR signaling has a crucial role in HBV-HCC shows the potential of this signaling pathway as a molecularly targeted therapy for this neoplasm. Research has established that cross talk occurs between the IGF-IR pathway and several other signaling pathways, suggesting the rationale for cotargeting IGF-IR and other receptor(s) simultaneously [19]. The clinical benefit of sorafenib, a multikinase inhibitor inhibiting at least Ras and Jak/Stat pathways, provided strong support for cotargeting therapy in clinical practice. Future targeted therapy for HCC should include agents inhibiting IGF-IR signaling, particularly for tumors induced by HBV. This may at least partially achieved by antagonist of inflammatory mediators in the tumor microenvironment.

## Supporting Information

S1 Fig Correlations of the levels of MCP-1/CD68 and pluripotent transcription factors OCT4 and NANOG in HBV-HCC or HBV-negative HCC tissues. (PDF)

S2 Fig Effects of inflamed-CM dilution on mRNA levels of *POU5F1* (*OCT4*), *NANOG*, and *IGF-IR* in HepG2.2.15 and Hep3B cells. (PDF)

S1 Table Real-time quantitative PCR primer and product size. (PDF)

S2 Table List of antibodies. (PDF)

## References

[1] Coussens LM, Werb Z. Inflammation and cancer. Nature. 2002;420: 860–867. 12490959 10.1038/nature01322 PMC2803035

[2] Berasain G, Castillo J, Perugorria MJ, Latasa MU, Prieto J, Avila MA. Inflammation and liver cancer. Ann NY Acad Sci. 2009;1155: 206–221. 10.1111/j.1749-6632.2009.03704.x 19250206

[3] Arndt W, Sandra K, Ina N, Henning S, Jochem K, Maria H, et al Trends in epidemiology, treatment, and survival of hepatocellular carcinoma patients between 1998 and 2009: an analysis of 1066 cases of a German HCC registry. J Clin Gastroenterol. 2014;48: 279–289. 10.1097/MCG.0b013e3182a8a793 24045276

[4] Yang JD, Nakamura I, Roberts LR. The tumor microenvironment in hepatocellular carcinoma: Current status and therapeutic targets. Semin Cancer Biol. 2011;21: 35–43. 10.1016/j.semcancer.2010.10.007 20946957 PMC3050428

[5] Ajani JA, Song S, Hochster HS, Steinburg IB. Cancer Stem Cells: The promise and the potential. Semin Oncol. 2015;42: S3–S17. 10.1053/j.seminoncol.2015.01.001 25839664

[6] Tanno T, Matsui W. Development and maintenance of cancer stem cells under chronic inflammation. J Nippon Med Sch. 2011;78: 138–145. 21720087 10.1272/jnms.78.138 PMC3380605

[7] Shigdar S, Li Y, Bhattacharya S, O'Connor M, Pu C, Lin J, et al Inflammation and cancer stem cells. Cancer Lett. 2014;345: 271–278. 10.1016/j.canlet.2013.07.031 23941828

[8] Csermely P, Hodsagi J, Korcsmaros T, Módos D, Perez-Lopez AR, Szalay K, et al Cancer stem cells display extremely large evolvability: Alternating plastic and rigid networks as a potential mechanism: Network models, novel therapeutic target strategies, and the contributions of hypoxia, inflammation and cellular senescence. Semin Cancer Biol. 2015;30: 42–51. 10.1016/j.semcancer.2013.12.004 24412105

[9] Jinushi M, Chiba S, Yoshiyama H, Masutomi K, Kinoshita I, Dosaka-Akita H, et al Tumor-associated macrophages regulate tumorigenicity and anticancer drug responses of cancer stem/initiating cells. Proc Natl Acad Sci USA. 2011;108: 12425–12430. 10.1073/pnas.1106645108 21746895 PMC3145680

[10] Ma N, Thanan R, Kobayashi H, Hamman O, Wishahi M, Leithy TE, et al Nitrative DNA damage and Oct3/4 expression in urinary bladder cancer with Schistosoma haematobium infection. Biochem Bioph Res Co. 2011;414: 344–349. 10.1016/j.bbrc.2011.09.073 21951846

[11] Knight B, Matthews VB, Akhurst B, Croager EJ, Klinken E, Abraham LJ, et al Liver inflammation and cytokine production, but not acute phase protein synthesis accompany the adult liver progenitor (oval) cell response to chronic liver injury. Immunol Cell Biol. 2005;83: 364–374. 16033531 10.1111/j.1440-1711.2005.01346.x

[12] He G, Dhar D, Nakagawa H, Font-Burgada J, Ogata H, Jiang Y, et al Identification of liver cancer progenitors whose malignant progression depends on autocrine IL-6 signaling. Cell. 2013;155: 384–396. 10.1016/j.cell.2013.09.031 24120137 PMC4015514

[13] Boyault S, Rickman DS, de Reyniès A, Balabaud C, Rebouissou S, Jeannot E, et al Transcriptome classification of HCC is related to gene alterations and to new therapeutic targets. Hepatology. 2007;45: 42–52. 17187432 10.1002/hep.21467

[14] Chang TS, Wu YC, Chi CC, Su WC, Chang PJ, Lee KF, et al Activation of IL6/IGF-IR confers poor prognosis of HBV-related hepatocellular carcinoma through induction of OCT4/NANOG expression. Clin Cancer Res. 2015;21: 201–210. 10.1158/1078-0432.CCR-13-3274 25564572

[15] Bendall SC, Stewart MH, Menendez P. George D, Vijayaragavan K, Werbowetski-Ogilvie T, et al IGF and FGF cooperatively establish the regulatory stem cell niche of pluripotent human cells in vitro. Nature. 2007; 448: 1015–1023. 17625568 10.1038/nature06027

[16] Wang L, Schulz TC, Sherrer ES, Dauphin DS, Shin S, Nelson AM, et al Self-renewal of human embryonic stem cells requires insulin-like growth factor-1 receptor and ERBB2 receptor signaling. Blood. 2007;110: 4111–4119. 17761519 10.1182/blood-2007-03-082586 PMC2190616

[17] Huang YH, Chin CC, Ho HN, Chou CK, Shen CN, Kuo HC, et al Pluripotency of mouse spermatogonial stem cells maintained by IGF-I-dependent pathway. FASEB J. 2009;23: 2076–2087. 10.1096/fj.08-121939 19246485

[18] Wang XQ, Ongkeko WM, Chen L, Yang ZF, Lu P, Chen KK, et al Octomer 4 (Oct4) mediates chemotherapeutic drug resistance in liver cancer cells through a potential Oct4–AKT–ATP-binding cassette G2 pathway. Hepatology. 2010;52: 528–539. 10.1002/hep.23692 20683952

[19] Desbois-Mouthon C, Baron A, Eggelpoel MB, Fartoux L, Venot C, Bladt F, et al Insulin-like growth factor-1 receptor inhibition induces a resistance mechanism via the epidermal growth factor receptor/HER3/AKT signaling pathway: rational basis for cotargeting insulin-like growth factor-1 receptor and epidermal growth factor receptor in hepatocellular carcinoma. Clin Cancer Res. 2009;15: 5445–5456. 10.1158/1078-0432.CCR-08-2980 19706799

[20] Ramanan V, Scull MA, Sheahan TP, Rice CM, Bhatia SN. New methods in tissue engineering: Improved models for viral infection. Annu Rev Virol. 2014;1: 475–499. 25893203 10.1146/annurev-virology-031413-085437 PMC4398347

[21] Chiao H, Foster S, Thomas R, Lipton J, Star RA. Alpha-melanocyte-stimulating hormone reduces endotoxin-induced liver inflammation. J Clin Invest. 1996;97: 2038–2044. 8621792 10.1172/JCI118639 PMC507277

[22] Tarao K, Takemiya S, Tamai S, Sugimasa Y, Ohkawa S, Akaike M, et al Relationship between the recurrence of hepatocellular carcinoma (HCC) and serum alanine aminotransferase levels in hepatectomized patients with hepatitis C virus-associated cirrhosis and HCC. Cancer. 1997;79: 688–694. 9024706 10.1002/(sici)1097-0142(19970215)79:4<688::aid-cncr5>3.0.co;2-a

[23] Matsumoto K, Yoshimoto J, Sugo H, Kojima K, Futagawa S, Matsumoto T. Relationship between the histological degrees of hepatitis and the postoperative recurrence of hepatocellular carcinoma in patients with hepatitis C. Hepatol Res. 2002;23: 196–201. 12076715 10.1016/s1386-6346(01)00180-2

[24] Cescon M, Cucchetti A, Grazi GL, Ferrero A, Viganò L, Ercolani G, et al Role of hepatitis B virus infection in the prognosis after hepatectomy for hepatocellular carcinoma in patients with cirrhosis. Arch Surg. 2009;144: 906–913. 10.1001/archsurg.2009.99 19841357

[25] Wu HC, Tsai HW, Teng CF, Hsieh WC, Lin YJ, Wang HC, et al Ground-glass hepatocytes co-expressing hepatitis B virus X protein and surface antigens exhibit enhanced oncogenic effects and tumorigenesis. Hum Pathol. 2014;45: 1294–1301. 10.1016/j.humpath.2013.10.039 24767856

[26] Sell S, Leffert HL. Liver cancer stem cells. J Clin Oncol. 2008;26: 2800–2805. 10.1200/JCO.2007.15.5945 18539957 PMC2515096

[27] Takahashi K, Yamanaka S. Induction of pluripotent stem cells from mouse embryonic and adult fibroblast cultures by defined factors. Cell. 2006:126: 663–676. 16904174 10.1016/j.cell.2006.07.024

[28] Shan J, Shen J, Liu L, Xia F, Xu C, Duan G, et al Nanog regulates self-renewal of cancer stem cells through the insulin-like growth factor pathway in human hepatocellular carcinoma. Hepatology. 2012;56: 1004–1014. 10.1002/hep.25745 22473773

[29] Chiba T, Kita K, Zheng YW, Yokosuka O, Saisho H, Iwama A, et al Side population purified from hepatocellular carcinoma cells harbors cancer stem cell–like properties. Hepatology. 2012;44: 240–251. 10.1002/hep.21227 16799977

[30] Tovar V, Alsinet C, Villanueva A, Hoshida Y, Chiang DY, Solé M, et al IGF activation in a molecular subclass of hepatocellular carcinoma and pre-clinical efficacy of IGF-IR blockage. J Hepatol. 2010;52: 550–559. 10.1016/j.jhep.2010.01.015 20206398 PMC3662876

[31] Llovet JM, Ricci S, Mazzaferro V, Hilgard P, Gane E, Blanc JF, et al Sorafenib in advanced hepatocellular carcinoma. N Engl J Med. 2008;359: 378–390. 10.1056/NEJMoa0708857 18650514

[32] Fransvea E, Paradiso A, Antonaci S, Giannelli G. HCC heterogeneity: molecular pathogenesis and clinical implications. Cell Oncol. 2009;31: 227–233. 10.3233/CLO-2009-0473 19478390 PMC4619048

[33] Villanueva A, Newell P, Chiang DY, Friedman SL, Llovet JM. Genomics and signaling pathways in hepatocellular carcinoma. Semin Liver Dis. 2007;27: 55–76. 17295177 10.1055/s-2006-960171

