# Correction: Inflammation Promotes Expression of Stemness-Related Properties in HBV-Related Hepatocellular Carcinoma

**DOI:** 10.1371/journal.pone.0171176

**Published:** 2017-01-26

**Authors:** Te-Sheng Chang, Chi-Long Chen, Yu-Chih Wu, Jun-Jen Liu, Yung Che Kuo, Kam-Fai Lee, Sin-Yi Lin, Sey-En Lin, Shui-Yi Tung, Liang-Mou Kuo, Ying-Huang Tsai, Yen-Hua Huang

The following grant numbers from the funder Chang Gung Memorial Hospital are missing from the Funding section: CMRPG6C0061, CMRPG6C0062, and CMRPG6C0063
