# Supplementary material for: Inflammation Promotes Expression of Stemness-Related Properties in HBV-Related Hepatocellular Carcinoma
Source: PLoS One. 2016 Feb 26;11(2):e0149897. doi: 10.1371/journal.pone.0149897 (PMC4769282; doi:10.1371/journal.pone.0149897)

Supporting information

S1 Fig. Correlations of the levels of MCP-1/CD68 and pluripotent transcription factors OCT4 and NANOG in HBV-HCC or HBV-negative HCC tissues.

(A) HBV-HCC, n = 57 (B) HBV negative (NBNC and HCV)-HCC, n = 53. Spearman's correlation.

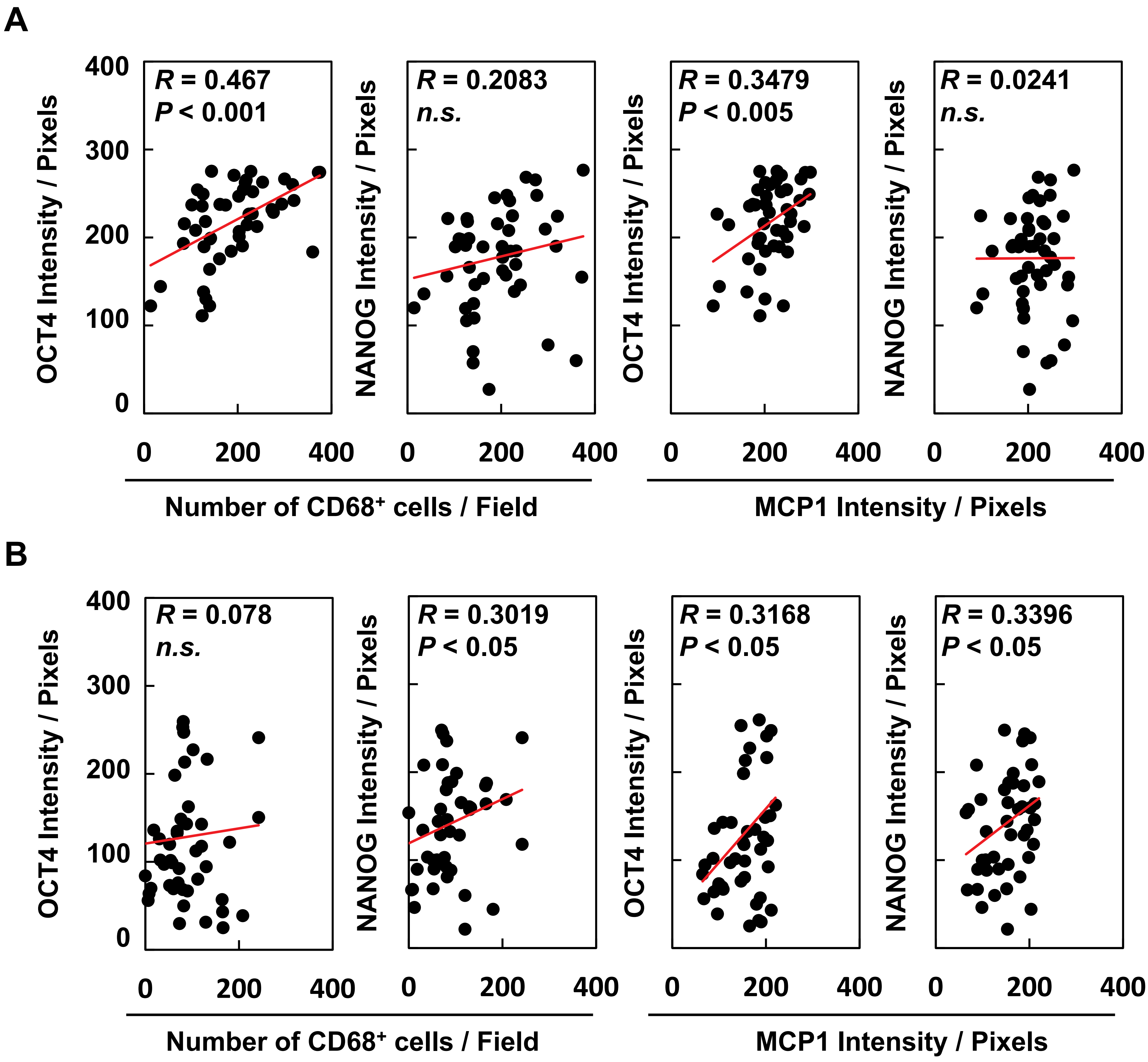

Supplement: S1 Fig — (PDF) [file pone.0149897.s001.pdf]
