# Supplementary material for: Inflammation Promotes Expression of Stemness-Related Properties in HBV-Related Hepatocellular Carcinoma
Source: PLoS One. 2016 Feb 26;11(2):e0149897. doi: 10.1371/journal.pone.0149897 (PMC4769282; doi:10.1371/journal.pone.0149897)

Supporting information

S2 Fig. Effects of inflamed-CM dilution on mRNA levels of *POU5F1* (*OCT4*), *NANOG*, and *IGF-IR* in HepG2.2.15 and Hep3B cells.

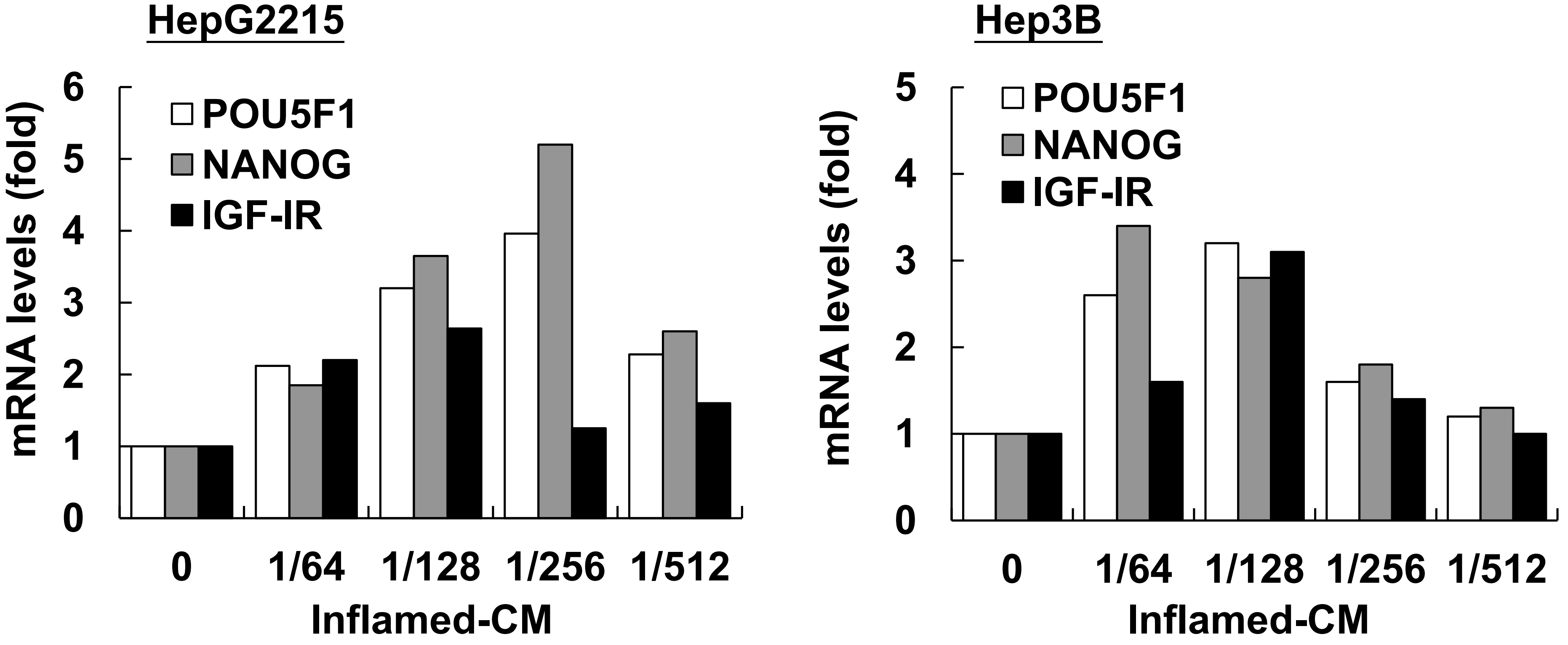

Supplement: S2 Fig — (PDF) [file pone.0149897.s002.pdf]
