# Supplementary material for: Inflammation Promotes Expression of Stemness-Related Properties in HBV-Related Hepatocellular Carcinoma
Source: PLoS One. 2016 Feb 26;11(2):e0149897. doi: 10.1371/journal.pone.0149897 (PMC4769282; doi:10.1371/journal.pone.0149897)
Supplement: S1 Table — (PDF) [file pone.0149897.s003.pdf]

S1 Table Real-time PCR primers and product size

Gene	Accession	Forward Primers	Reverse Primers	Annealing Temp (°C)	Product Size (bp)
OCT4	NM_002701.4	5’-CAACTCCGATGGGGCCT-3’	5’-CTTCAGGAGCTTGGCAAATTG-3’	53	148
NANOG	NM_024865.2	5’-CCTGTGATTTGTGGGCCTG -3’	5’-GACAGTCTCCGTGTGAGGCAT-3’	53	78
SOX2	NM_003106.3	5’-GTATCAGGAGTTGTCAAGGCAGAG-3’	5’-TCCTAGTCTTAAAGAGGCAGCAAA-3’	57	79
IGF-I	NM_000618.3	5’-CTGCTTCCGGAGCTGTGATCTGAG-3’	5’-TCCTTCTGAGCCTTGGGCATGTC-3’	55	97
IGF-IR	NM_000875.3	5’-CTCCTGTTTCTCTCCGCCG-3’	5’-ATAGTCGTTGCGGATGTCGAT-3’	54	85
β-2M	NM_004048.2	5’-GTCTCGCTCCGTGGCCTTA-3’	5’-TGAATCTTTGGAGTACGCTGGATA-3’	56	81
