# Supplementary material for: Inflammation Promotes Expression of Stemness-Related Properties in HBV-Related Hepatocellular Carcinoma
Source: PLoS One. 2016 Feb 26;11(2):e0149897. doi: 10.1371/journal.pone.0149897 (PMC4769282; doi:10.1371/journal.pone.0149897)
Supplement: S2 Table — (PDF) [file pone.0149897.s004.pdf]

S2 Table List of antibodies

Protein	Assay	Ab Cat. No.	Company	Origin	Dilution	Incubation Period
OCT4	IHC	sc-5279	Santa Cruz Biotech	mouse	1:100	overnight, 4°C
NANOG	IHC	ab21624	Abcam	rabbit	1:100	overnight, 4°C
MCP1	IHC	sc-1784	Santa Cruz Biotech	goat	1:200	overnight, 4°C
CD68	IHC	Ab955	Abcam	mouse	1:200	overnight, 4°C
Control IgG	IHC	#76870	Jackson ImmunoResearch	rabbit		overnight, 4°C
OCT4	WB	sc-5279	Santa Cruz Biotech	mouse	1:1000	overnight, 4°C
NANOG	WB	RCAB002P-F	ReproCell	rabbit	1:1000	overnight, 4°C
p-IGF-IR	WB	sc-101703	Santa Cruz Biotech	rabbit	1:1000	overnight, 4°C
IGF-IRβ	WB	sc-713	Santa Cruz Biotech	rabbit	1:1000	overnight, 4°C
p-AKT (Ser473)	WB	#9271	Cell Signaling	rabbit	1:1000	overnight, 4°C
AKT	WB	sc-8312	Santa Cruz Biotech	rabbit	1:200	overnight, 4°C
EGFP	WB	sc-9996	Santa Cruz Biotech	mouse	1:200	overnight, 4°C
N-CADHERIN	WB	610921	BD	mouse	1:2000	overnight, 4°C
SLUG	WB	#9585	Cell Signaling	rabbit	1:1000	overnight, 4°C
β-Actin	WB	sc-47778	Santa Cruz Biotech	mouse	1:1000	overnight, 4°C

WB, western blot; IHC, immunohistochemistry.
